# Bond Polarizability as a Probe of Local Crystal Fields in Hybrid Lead-Halide Perovskites

**DOI:** 10.1021/acs.jpclett.3c01158

**Published:** 2023-07-05

**Authors:** Yujing Wei, Artem G. Volosniev, Dusan Lorenc, Ayan A. Zhumekenov, Osman M. Bakr, Mikhail Lemeshko, Zhanybek Alpichshev

**Affiliations:** † Institute of Science and Technology Austria (ISTA), Am Campus 1, 3400, Klosterneuburg, Austria; ¶ KAUST Catalysis Center (KCC), Division of Physical Sciences and Engineering, King Abdullah University of Science and Technology (KAUST), Thuwal, 23955-6900, Kingdom of Saudi Arabia

## Abstract

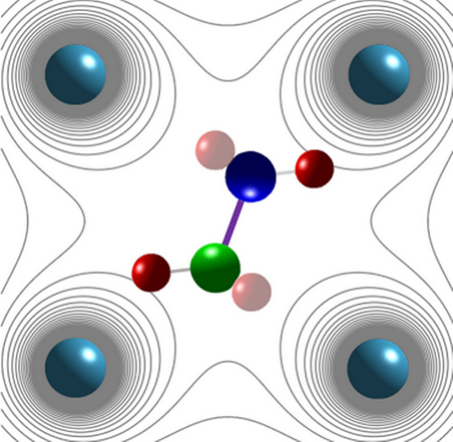

A rotating organic cation and a dynamically disordered soft inorganic cage are the hallmark features of organic-inorganic lead-halide perovskites. Understanding the interplay between these two subsystems is a challenging problem, but it is this coupling that is widely conjectured to be responsible for the unique behavior of photocarriers in these materials. In this work, we use the fact that the polarizability of the organic cation strongly depends on the ambient electrostatic environment to put the molecule forward as a sensitive probe of the local crystal fields inside the lattice cell. We measure the average polarizability of the C/N–H bond stretching mode by means of infrared spectroscopy, which allows us to deduce the character of the motion of the cation molecule, find the magnitude of the local crystal field, and place an estimate on the strength of the hydrogen bond between the hydrogen and halide atoms. Our results pave the way for understanding electric fields in lead-halide perovskites using infrared bond spectroscopy.

The efficiency of hybrid organic-inorganic lead-halide perovskite (HOIP)-based solar cells has recently nearly reached the levels of the state-of-the-art conventional Si-based devices.^1,2^ On the microscopic level this impressive performance is contingent upon the presence of charged photocarriers that can travel unimpeded sufficiently far to reach the contacts of the photovoltaic element. The fact that this is indeed the case for lead-halide perovskites,^3−8^ despite much higher defect concentration in HOIP samples as compared to standard photovoltaic materials such as Si,^9^ is arguably the biggest puzzle in the field of perovskite research. While an exhaustive explanation is still lacking, the unexpectedly high efficiency of HOIPs, be it in photocarrier separation^10^ or “neutralization” of defect centers,^11^ suggests the pre-eminent role of charge screening. One possible source of such screening pointed out early on is the quasi-freely rotating polar organic cation occupying the A-site of HOIPs such as CH_3_NH_3_^+^ (methylammonium, MA),^12−15^ while others argue for the inorganic cage as the main responsible party.^16^ In order to bring more clarity to this problem, a detailed understanding of the interaction between cation molecules and the surrounding cage is necessary. Unsurprisingly, this is not an easy task as while the former is semi-independent, the latter is soft, dynamically disordered, and highly anharmonic^17−22^ with significant ionic conductivity^23^ to the extent of being dubbed “plastic crystals” in some works.^24,25^

The very fact that the A-site cation retains its chemical autonomy inside the cage,^29−31^ implies that the interaction between the two is mostly of an electrostatic nature.^32^ To characterize the magnitude of this coupling, one needs therefore to be able to probe the local electric fields within the crystal, primarily the ones experienced by hydrogen atoms. Unfortunately, conventional tools such as nuclear quadrupole resonance spectroscopy^33−35^ are not particularly suitable for this purpose as the lightest nuclei (proton, deuteron) have either no or small quadrupolar moment. In this light, we draw attention to the fact that in HOIPs, the A-site cation can itself act as a sensitive local probe of its immediate environment. Indeed, on the one hand, the cation is chemically decoupled from the inorganic cage and can be treated as a separate molecule, and on the other hand, it is often simple enough to allow for an exhaustive theoretical treatment.

In this work, we show that the ambient electrostatic environment can noticeably modify basic properties of the molecule such as its dynamic susceptibility using methylammonium in MAPbBr_3_ as an example. We compare these theoretical results to the measured infrared polarizability of methylammonium, draw conclusions about the character of coupled cation-cage dynamics, put a numeric value on the effective local electric field experienced by the hydrogen atoms in methylammonium cation and estimate the strength of the hydrogen bond between H and Br atoms. These findings pave the way for employing infrared probes^36,37^ for studying organic-inorganic crystal structures such as HOIPs.

Polarizability α_*ij*_ is a basic physical property of a molecule that relates the induced dipole moment, *p⃗*, of a molecule to the applied electric field, *E⃗*, as *p*_*i*_(*ω*) = α_*ij*_(*ω*)*E*_*j*_(*ω*). Microscopically, *p⃗* can be induced either by electron cloud polarization or by deformation of the molecule in question. When the field *E⃗* becomes comparable to the interatomic one, the linear relation between *p⃗* and *E⃗* above is no longer valid and should be amended by assuming that *α_ij_* depends on *E⃗*.^38^ Such fields are natural for intracell lattice environments, meaning that even if the molecule retains its chemical autonomy within a compound, one should expect its vibrational polarizability to be noticeably affected by the local fields inside a lattice unit. This turns the polarizability of the molecule into a marker of these fields, which can be investigated in linear optical experiments, where probing fields are weak by definition.

Any molecular deformation can be decomposed into normal vibration modes,^39^ where each normal mode *k* has a specific resonance frequency Ω_*k*_ and can be ascribed a frequency-dependent polarizability tensor *α*_*ij*_^*k*^(*ω*). The sum of these terms determines the refractive index *n*(*ω*) of the medium; the contribution of each normal mode to *n* is the most prominent near its resonant frequency Ω_*k*_.^40^ In this work, we choose to focus on the vibration modes of methylammonium that correspond to the longitudinal displacement of the hydrogen atoms along the direction of the bond, *n⃗*, to the nearby N or C atoms (see Figure 1A,B). The main reason for this choice is that these modes are by far the strongest in terms of IR-intensity.^17,30,41^ Elementary counting of the degrees of freedom reveals (in total) 6 modes that involve longitudinal stretching of the C(N)–H bond, which will be labeled as *H*_*k*_ or simply *H*.

**Figure 1:**
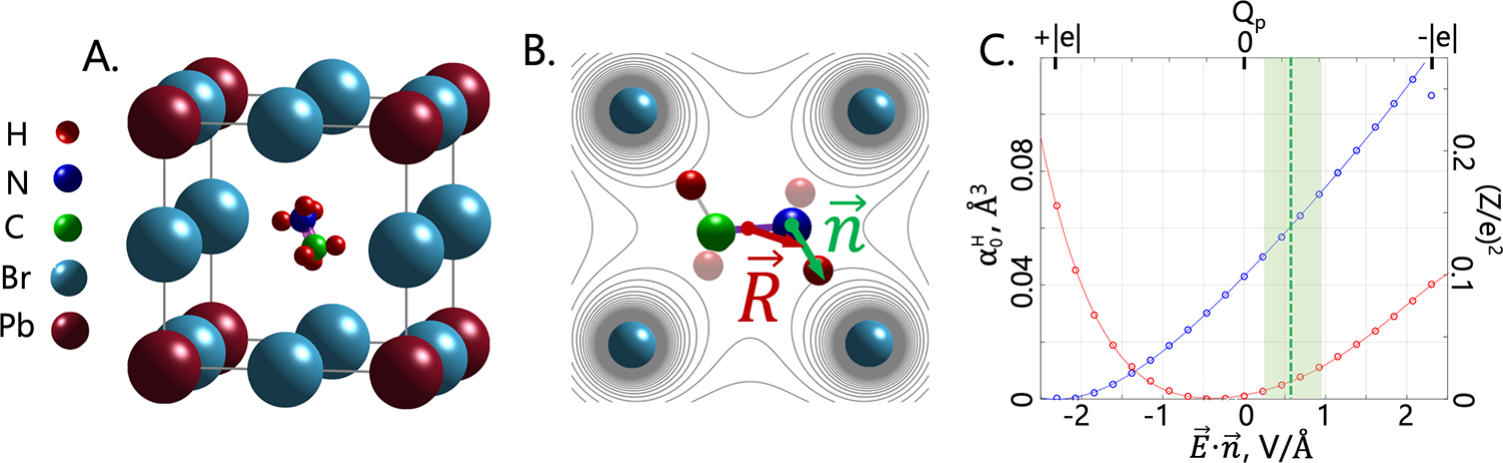
(A) Lattice structure of MAPbBr_3_ (to scale). (B) Cartoon of the MA cation in the electrostatic field of the inorganic cage (not to scale); *n⃗* is the unit vector along the bond direction; *R⃗* is the distance to the center of the bond from the center of the lattice unit. (C) Static longitudinal polarizabilities *α*_0_^*H*^ of C(N)–H bonds as a function of the electric field at the locus of the bond. For comparison, the field is also parametrized by the value of a point charge *Q*_p_ placed on the line connecting one of the H atoms to the neighboring N(C) atom, at a distance *d* = 2.5 Å from H. This *d* value corresponds to the most stable H–Br separation in the cubic phase of MAPbBr_3_ for the N–H hydrogen^26,27^ (for consistency, the same distance is chosen for C–H in our calculations). α_0_^*H*^ is also parametrized with a square of the effective (Born) charge of an H atom, *Z*, as α_0_^*H*^ = *Z*^2^/*m*_H_Ω_*H*_^2^ (see ref (28)), where *m*_H_ is the mass of a hydrogen atom, and Ω_*H*_ is the resonant frequency of the C(N)–H stretching mode. The green dashed line (green rectangle) marks the estimated value (error margin) of the crystal field experienced by the C(N)–H bond (see the text for details).

The simplicity of methylammonium allows for a direct computation of the polarizability of the C(N)–H stretching mode, *α*_0_^*H*^, by means of density functional theory (DFT); for more details see ref (28). In Figure 1C we show how the (longitudinal) polarizabilities of the C–H and N–H bonds depend on the strength of the longitudinal electric field. To give these values some intuitive sense, the applied electric field at the locus of the C(N)–H bond is also parametrized by the charge *Q*_p_ of a point source located on the C(N)–H line at a distance *d* = 2.5 Å from the H atom; the value for *d* is motivated by the H–Br distance at room temperature.^26,27,42^ Although, the present work focuses on the stoichiometric case, we note that our calculations indicate that the effect of localized lattice defects such as vacancies or color centers must be very significant. Indeed, the associated fields are on the order of *E*_def_ ∼ |*e*|/*d*^2^ ∼ 1 V/Å in the vicinity of the molecule. They can renormalize bond polarizability by about 100%, meaning that the methylammonium cation can be a very sensitive probe of lattice imperfections.

The microscopic longitudinal bond polarizability α_*ij*_^*H*^ depends on the fluctuating local crystal field  (see Figure 1C). To relate it to the polarizability ⟨*α^H^*⟩ measured in experiment, *α*_*ij*_^*H*^ must be averaged over all spatial orientations of the bond and different configurations of . For MAPbBr_3_, which is cubic (on average) at room temperature, one can write (see ref (28)):
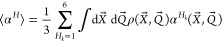
1where the index *H*_*k*_ runs over the six longitudinal C(N)–H stretching modes (note that we assume that the polarizability of the molecule is the sum of polarizabilities of its bonds^43,44^); X⃗  are the positions of atoms in the molecule (the cage);  is the density matrix that determines probabilities of a specific lattice-molecule configuration given by *X⃗* and *Q⃗*;  is the longitudinal polarizability of the *H*_*k*_th bond in a configuration  (here we rely on the Born–Oppenheimer approximation to assume that  only depends on instantaneous positions of atoms in the cage). To derive eq 1, we noted that according to our DFT calculations, , where n⃗ is a unit vector along the bond direction, see ref (28) for further details.

The quantities *ρ* and *α^H^* in eq 1 can be determined theoretically from the literature and DFT calculations for different scenarios, such as the presence of defects. Equation 1 relates these theoretical calculations to the measured average polarizability, ⟨*α^H^*⟩, allowing one to investigate microscopic properties of HOIPs using molecules as experimental probes.

To illustrate the formal discussion above, we proceed with measuring the average polarizability ⟨*α*^*H*^⟩ for a bulk single crystal sample of MAPbBr_3_ in the cubic phase. For this purpose, we measure the group refractive index *n*_g_(*ω*) in the mid-infrared frequency range relevant for vibrational degrees of freedom of methylammonium.^17^ The advantage of the group index for probing molecular polarizability, which in general provides much weaker contribution to the refractive index as compared to the electronic one, lies in the fact that *n*_g_(*ω*) features stronger divergence near resonances and is therefore more sensitive as compared to the phase index *n*_ph_(*ω*). Indeed, *n*_g_(*ω*)/*n*_ph_(*ω*) ≈ Ω/(*ω* – Ω) near the resonance frequency Ω (see the inset in Figure 2A).

**Figure 2:**
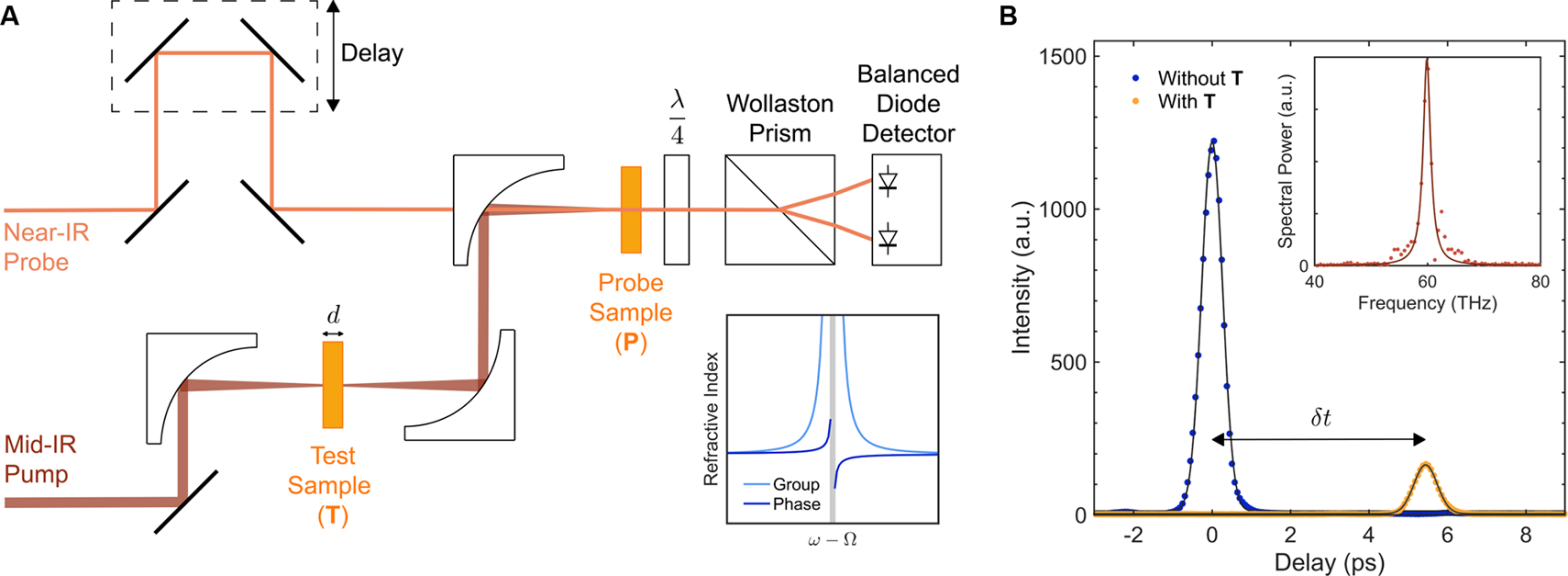
(A) Optical Kerr effect-based pump-probe method to obtain the group refractive index by measuring the time that it takes for a pulse to go through the test sample. The delay between the mid-IR pump and the near-IR probe is measured using balance detection of the mid-IR induced birefringence in the probe sample. The inset shows the region of anomalous dispersion for the phase and group refractive indexes, showing that the group refractive index is more divergent. (B) Example of measured signals of the pump-probe delay with and without test sample. The inset shows Fourier-transform infrared spectroscopy (FTIR) of the pump pulse.

To measure the group refractive index in a direct manner, we develop a time-resolved setup depicted in Figure 2A. The setup utilizes two samples of single-crystal MAPbBr_3_: the actual test sample (**T** in Figure 2) in which *n*_g_ is measured and a probe sample for a broadband ultrafast detection of mid-infrared pulses by means of the optical Kerr effect (**P** in Figure 2). By measuring the time of arrival of the mid-IR pulse at the probe sample with and without the test sample, one obtains the delay *δt* introduced by the test sample. The time delay *δt* for the mid-IR pulse due to traveling through the perovskite sample of thickness *d* = 1.6 mm, is , where *n*_g_ and *n*_g_^air^ ≈ 1 are the group refractive indices of MAPbBr_3_ and air, respectively; *c* is the speed of light.

The ultrafast optical Kerr effect in the probe sample is detected by the standard time-resolved pump-probe method in a balanced detection scheme (see Figure 2A and ref (28)). An example of a time-resolved transient Kerr response for *λ* = 5 μm mid-IR pump with and without the test sample is depicted in Figure 2B. The difference in arrival time of the mid-IR pulse at the probe sample *δt* is measured as the distance between the two peak positions; the inset shows the FTIR of the pump pulse (see ref (28)). Performing similar measurements for other mid-IR wavelength values, one can extract the perovskite group index *n*_g_(*ω*) = *n*_g_^air^ + *cδt*/*d* over a broad range of wavelengths.

The measured group refractive index dispersion *n*_g_(*ω*) of bulk single crystal MAPbBr_3_ as a function of the photon energy, *ℏω*, is displayed in Figure 3. Here, we identify two regions of absorption, one in the low energy region at ∼0.17 eV (1400 cm^–1^) and another at a higher energy ∼0.37 eV (3000 cm^–1^), which can be associated with the bending and stretching modes of C(N)–H bonds, respectively.^17,30,41^ The low value of extinction, *κ*, (see ref (28)) in the region outside the immediate vicinity of the absorption band corresponding to the C(N)–H stretching modes justifies our assumption that these modes can be approximated as isolated from the nearby C(N)–H bending modes.

**Figure 3:**
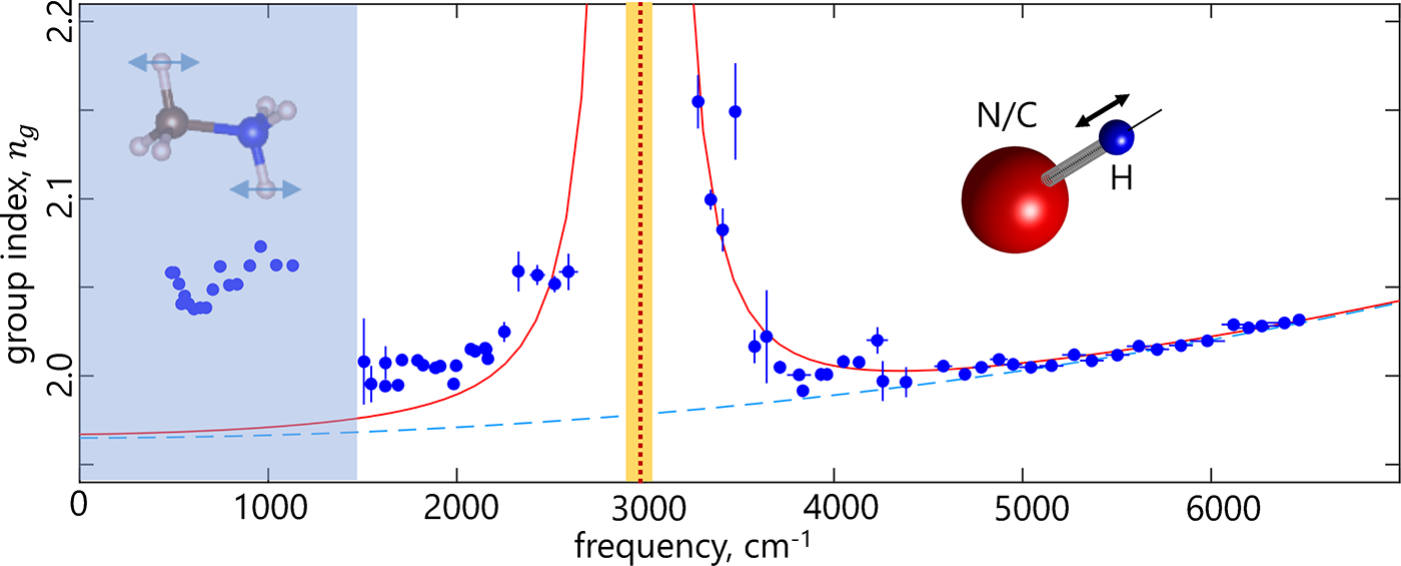
Group refractive index *n*_g_ measured using the setup depicted in Figure 2. The horizontal error bars are the full width at half-maximum (FWHM) of the FTIR calibration, and the vertical error bars are the FWHM of the dispersion from Figure 2B. The low-frequency region of vibrational resonances is indicated by the blue shading. Sketch of the corresponding (bending) molecular modes is presented in the shaded region. The right inset is an illustration of a longitudinal C(N)–H bond stretching, representing the entire cluster of 6 stretching modes in the energy region around 3000 cm^–1^. The solid red line is the fit according to eq 2; the vertical red dotted line marks the resonant frequency, Ω_*H*_, from the fit; the light blue dashed curve is the electronic group index obtained from the Sellmeier fit to the high energy data of ref (45); the yellow shaded region illustrates the frequency spread within the longitudinal-stretching mode cluster.^17,30,41,46^

To extract the molecular contribution to the refractive index, we need to subtract the electronic contribution from the measured infrared *n*_g_(*ω*). (Note that the electronic contribution to the refractive index is typically the dominant one, since electrons are much lighter than ions. Only close to a molecular resonance does the molecular contribution become important.) To this end, we use our previous measurements of the phase index in the visible and near-IR region.^45^ In this high frequency range near the bulk band transition frequency, *ℏω* ∼ Δ_gap_ and the refractive index is determined by the electronic polarizability of the lead-halide cage. By first fitting the high-frequency phase index from ref (45) with a Sellmeier expression, and then calculating the group index from it and extrapolating the result to the mid-IR frequencies, one can obtain the electronic part *n*_g,el_(*ω*) of the total group refractive index *n*_g_(*ω*) coming from the crystal cage. *n*_g,el_(*ω*) in the mid-IR range calculated this way is plotted as a light blue dashed line in Figure 3 providing an excellent fit to the data in the relevant frequency range away from the nearby absorption band. The molecular part of the group index *n*_g,mol_ is found by subtracting the cage electronic contribution from the total refractive index: *n*_g,mol_(*ω*) = *n*_g_(*ω*) – *n*_g,el_(*ω*) (see ref (28)).

Once *n*_g,mol_(*ω*) is known, it is possible to calculate the polarizability of the given mode by fitting it to the classical resonance profile near the resonant frequency. We focus on the C(N)–H stretching modes centered around *ℏ*Ω_*H*_ ≈ 0.37 eV (3000 cm^–1^). Note that the resonance in Figure 3 corresponds not to a single mode but instead to a group of 6 different modes involving stretchings of different C(N)–H bonds. However, due to strong mass mismatch between H and C(N), the natural frequencies of these modes are very similar.^17,30,41^ If we assume that C and N are infinitely heavy compared to hydrogen, then each vibration of H along the C(N)–H bond can be considered independent and decoupled from the rest. Within this approximation (that introduces an error of the order ∼*m*_H_/*m*_C_ ≈ 10%), we shall use an oscillator model with a single resonant frequency (see the inset in Figure 3).

To connect *n*_g,mol_(*ω*) to ⟨*α*_0_^*H*^⟩, we account for the screening effect due to the polarizable cage and write in the vicinity of Ω_*H*_ (see ref (28) for derivation),
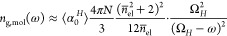
2where  is the electronic contribution of the phase refractive index at Ω_*H*_, and ⟨*α*_0_^*H*^⟩ is the average static (longitudinal) polarizability of the C(N)–H bond. The solid line in Figure 3 is the result of the fit of *n*_g,mol_(*ω*) to the expression in eq 2. From the fit, we obtain ⟨*α*_0_^*H*^⟩ ≈ (7.0 ± 1.1) × 10^–2^ Å^3^, with the error representing the 95% confidence range. Since the fitting error significantly exceeds the spread in resonant frequency values for C(N)–H stretching modes (∼10%, see above) one can neglect the latter for the sake of discussion.

With an experimental value for ⟨*α*^*H*^⟩ at hand, we compare it with that of eq 1 to see what can be said about the interaction between MA and the surrounding cage. In the simplest case, one may note that in some molecular dynamics studies the C–N axis of the cation appears to explore the available space quasi-uniformly^47,48^ and naively assume that the molecule rotations are uncorrelated with the inorganic cage. In terms of eq 1, this limit corresponds to , for which (see ref (28)) the average polarizability remains unrenormalized even in the presence of strong crystal fields 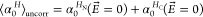
where *α*_0_^*H*^(*E* = 0) can be taken from Figure 1C so that  Å^3^. Since the discrepancy between this value and the experimental one above is beyond the error margin of our approach, we can conclusively rule out uncorrelated molecule-cage dynamics, in agreement with prior results.^26,49−51^ If, conversely, we fix the density matrix in eq 1 in accordance with previous reports in the literature,^28^ then we arrive at the value for the local crystal field experienced by the N–H bond |*E*_CF_| = (0.6 ± 0.3) V/Å by comparing the polarizabilities in Figure 1C with experimental values.

Note that in a stoichiometric lattice the Coulombic fields coming from the atoms, if treated as point sources, cancel each other very finely inside the lattice cell (see ref (28)). Therefore, to account for the relatively large value of *E*_CF_, we need to go beyond the point-source approximation for the atoms in the inorganic cage. The lowest-order multipole moment would be the quadrupolar moment *D* of the bromine atom with the axis along Pb–Br–Pb. Knowing |*E*_CF_| and the distance between H and Br one can estimate that *D* ∼ +1 eÅ^2^. Recalling now that a hydrogen bond can be defined as an interaction between H and a multipole potential of a neighboring atom,^52^ we estimate it from the energy of the H–Br electrostatic interaction  eV (for more details, see ref (28)). This value is in reasonable agreement with previous estimates^50,53^ of the energy of the H–Br hydrogen bond, illustrating that the proposed here molecular probe can provide important insight into the existence of hydrogen bonding in HOIPs.^54,55^

In conclusion, we propose the semiautonomous A-site cations in HOIPs as a sensitive local probe for optical spectroscopy, which complements the existing techniques such as NMR and NQR. To illustrate the formulated theoretical framework, we have analyzed the average polarizability of the N–H stretching mode extracted by measuring the group refractive index of a bulk single crystal sample of MAPbBr_3_ in the mid-IR wavelength range. Based on the analysis, we have ruled out the possibility of an uncorrelated motion of methylammonium cation in a PbBr cage and estimated local electric fields at the locus of the C(N)–H bond. We also estimated the value of the quadrupole moment for the Br atom, and the energy of the H–Br hydrogen bond. Our work proposes a new approach to the study of the complex behavior of the dynamically disordered inorganic cage, which will provide novel insight into fundamental properties of lead-halide perovskites. In particular, being all-optical, our approach can be employed to study ultrafast transient behavior of lattice irregularities, for example polaronic structures formed around photoexcitations. Since it is widely expected that the formation of such polarons underlies some most important optoelectronic properties of lead halide perovskites,^10^ our results offer an approach to clarify some of the most pressing questions related to the solar energy harvesting applications of lead-halide perovskites.

## Supporting Information Available

Additional experimental and theoretical details including description of methods. Derivation of equations and quantities discussed in the main text (PDF)
