# Supplementary material for: Bond Polarizability as a Probe of Local Crystal Fields in Hybrid Lead-Halide Perovskites
Source: J Phys Chem Lett. 2023 Jul 5;14(27):6309–14. doi: 10.1021/acs.jpclett.3c01158 (PMC10350961; doi:10.1021/acs.jpclett.3c01158)
Supplement: Supplementary file 1 — jz3c01158_si_001.pdf [file jz3c01158_si_001.pdf]

Supplementary Information: 'Bond Polarizability as a Probe of Local Crystal Fields in Hybrid Lead-Halide Perovskites'

Yujing Wei,[†,‡,§] Artem G. Volosniev,[†,‡] Dusan Lorenc,[†] Ayan A. Zhumekenov,[¶,‖] Osman M. Bakr,[¶] Mikhail Lemeshko,[†] and Zhanybek Alpichshev[*,†]

†*Institute of Science and Technology Austria (ISTA), Am Campus 1, 3400 Klosterneuburg, Austria*

‡*Equal contribution*

¶*KAUST Catalysis Center (KCC), Division of Physical Sciences and Engineering, King Abdullah University of Science and Technology (KAUST), Thuwal 23955-6900, Kingdom of Saudi Arabia*

§*Current address: Department of Chemistry, Columbia University, New York, New York 10027, United States*

‖*Current address: School of Materials Science and Engineering, Nanyang Technological University, 50 Nanyang Avenue, 639798 Singapore*

E-mail: alpishev@ist.ac.at

1. Sample Preparation

High quality bulk single crystal samples of $MAPbBr_3$ were grown by the inverse temperature crystallization method as described previously[1,2].

Chemicals.— CH_3NH_3Br (>99.99%) was purchased from GreatCell Solar Ltd. (formerly Dyesol) and used as received. $PbBr_2$ ($\geq$98%), DMF (anhydrous, 99.8%), and DMSO (anhydrous, $\geq$99.9%) were purchased from Sigma Aldrich and used as received.

Synthesis of $MAPbBr_3$ perovskite single crystals.—A 1.5 M solution of $CH_3NH_3Br/PbBr_2$ in DMF was prepared, filtered through 0.45-μm-pore-size PTFE filter; and the vial containing $0.5-1$ ml of the solution was placed on a hot plate at 30°C. Then the solution was gradually heated to ~ 60°C and kept at this temperature until the formation of $MAPbBr_3$ crystals. The crystals can be grown into larger sizes by elevating the temperature further. Finally, the crystals were collected and cleaned using a Kimwipe paper.

2. Calculation of bond polarizability

DFT Methods. The Born charges of H ions in the methylammonium were calculated by density functional theory (DFT) using the ORCA software package[3]. The parameters used for the calculation are as follows: B3LYP functional[4,5] and aug-cc-pVTZ[6,7] basis set for all steps of the calculations.

Bond polarizability. Since molecular polarizability is a dynamic property and every vibrational mode can be assigned its own contribution polarizability tensor, the focus of this work is on the most polarizable and therefore the most relevant modes. In the case of the methylammonium cation in the A-site of $CH_3NH_3PbBr_3$ hybrid lead-halide perovskite, by far the strongest modes in terms of IR-intensity are those that correspond to displacement of the hydrogen atoms along the direction of the bond to the nearby C or N atom[8–10]. Elementary counting of the degrees of freedom reveals that there are 6 modes that involve longitudinal stretching of the C(N)–H bond, but since both C and N atoms are much heavier than a proton, all of these modes are almost degenerate: $\Delta\Omega/\Omega_0 \sim m_{\mathrm{H}}/m_{\mathrm{C}} \approx 10\%$ is the error induced to our harmonic oscillator model with one resonant frequency Ω_H. We calculate the polarizability of such modes using the Born effective charge. The relationship is as follows (see the discussion around Eq. (S6) for more details):

$$\alpha_0^H = \frac{Z^2}{m_{\mathrm{H}}\Omega_H^2} \tag{S1}$$

where α_0^H is the static longitudinal polarizability of C(N)–H bonds, Ω_H is the resonant frequency of the relevant cluster of modes that correspond to longitudinal bond stretching, and Z is the Born effective charge, which is relatively simple to compute – we refer to previous studies[11] that calculate polarizability in a similar way.

To remind, Born effective charge Z of a given ion is a tensor defined as

$$Z_{ij} = \frac{\mathrm{d}p_i}{\mathrm{d}r_j}, \tag{S2}$$

where p_i is the total dipole moment of the molecule and r_j is the coordinate of the ion in question. In our case the polarizability of the C(N)–H bond stretching is related to the specific terms of the full Born charge tensor that correspond to the displacements of H ions along the corresponding bonds. In this work we calculate this "longitudinal" Born effective charge by following its definition above. Specifically, we first begin by finding the equilibrium atomic positions in the $CH_3NH_3^+$ ion by performing an initial geometry optimization and calculating the dipole moment of the resultant configuration. Then we displace the H ion in question by $\delta a = 0.01a_0$ in the direction of C(N), where a_0 is the equilibrium C(N)–H bond length. In the new configuration we re-calculate the total dipole moment $\vec{p}$ of the distorted molecule. An important detail here is that since net charge of methylammonium is not zero, we take care to keep the origin unmoved relative to the unperturbed atomic positions when re-calculating $\vec{p}$. Provided the origin is kept fixed, the change $\delta\vec{p} = \vec{p} - \vec{p}_0$ does not depend on it, and the Born effective charge corresponding to the "longitudinal" displacement of H along the C(N)–H bond is found as $Z = \delta\vec{p}/\delta a$.

To confirm that the chosen distortion δa is appropriate for determining Z, we plot in Fig. S1 the change of the dipole moment as a function of δa. We observe a linear dependence within the selected range, which confirms that Z is a well-defined physical property.

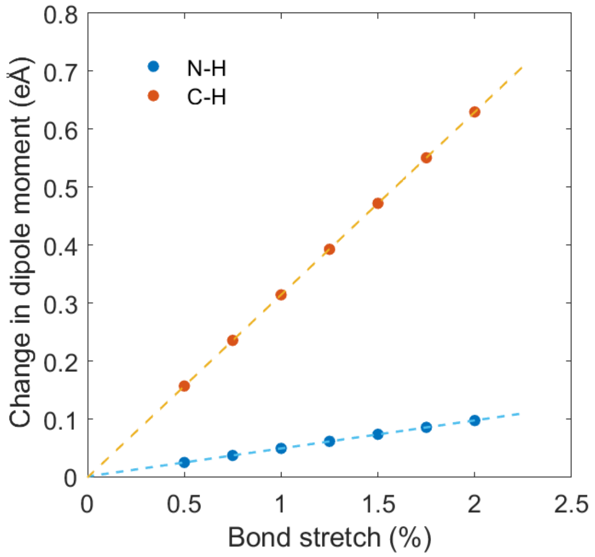

Figure S1: Difference in dipole moment of methylammonium molecule upon a slight stretch in one C(N)-H bond from the equilibrium geometry. The bond stretch is presented as a percentage of the equilibrium C(N)-H bond length a_0 and is proportional to δa. The dashed lines are linear fits to the data, where the coefficient of determination $R^2 = 1.00$ for both N-H and C-H stretch.

3. Derivation of Eq. (1) of the main text

In an external field, the dipole moment of a bond in a molecule changes as

$$\Delta p_{\parallel} = \alpha_{\parallel\perp} E_{\perp} + \alpha_{\parallel\parallel} E_{\parallel}; \qquad \Delta p_{\perp} = \alpha_{\perp\perp} E_{\perp} + \alpha_{\perp\parallel} E_{\parallel}. \tag{S3}$$

Here, $\parallel$ and $\perp$ determine the directions with respect to the C(N)–H axis of the bond. Note that there are two directions perpendicular to the bond – the notation $\perp$ is used for both, for convenience. We are interested in the polarizability $\alpha_{\parallel\parallel}$, which is denoted as $\langle \alpha_0^H \rangle$ in the main text.

To relate $\langle \alpha_0^H \rangle$ to the density matrix ρ (cf. Eq. (1) of the main text), we set $\alpha_{\parallel\perp}$ to zero, in agreement with our DFT calculations. [To have a physical picture of this observation, one should consider the C(N)–H bond as a charged ellipsoid, see, e.g.,[12].] Then, we write the 'longitudinal' part of the dipole moment of the molecule that is induced by a weak electric

field (assuming that the center of mass is not changed):

$$\Delta\vec{p}_{\parallel} = \int \mathrm{d}\vec{X}\mathrm{d}\vec{Q}\rho(\vec{X},\vec{Q}) \sum_{H_k} \vec{n}_k(\vec{X},\vec{Q}) Z_k(\vec{X},\vec{Q}) \Delta X_k, \tag{S4}$$

where the sum is over all C-H and N-H bonds; $\vec{n}_H$ is the unit vector that determines the direction of the bond, Z is the associated charge. The change in the position of the H_k atom along the bond, ΔX_k, is calculated using the harmonic approximation

$$\frac{\partial\left(m_{\mathrm{H}}\Omega_H^2 X_k^2/2 - X_k Z_k(\vec{E}\cdot\vec{n}_k)\right)}{\partial X_k} = 0 \;\rightarrow\; \Delta X_k = \frac{Z_k}{m_{\mathrm{H}}\Omega_H^2}\vec{E}\cdot\vec{n}_k, \tag{S5}$$

where m_{H} is the mass of a hydrogen atom. Note that we have assumed here that C and N atoms are infinitely heavy. This is done in line with the set of approximations made during the analysis of the data in the main text.

Let us now consider the quantity $\Delta\vec{p}_{\parallel}\cdot\vec{E}$. As the HOIP has (on average) a cubic lattice, $\Delta\vec{p}_{\parallel}\cdot\vec{E} = \langle\alpha_0^H\rangle E^2$, which allows us to write

$$\langle\alpha_0^H\rangle E^2 = \int \mathrm{d}\vec{X}\mathrm{d}\vec{Q}\rho(\vec{X},\vec{Q}) \sum_{H_k} (\vec{E}\cdot\vec{n}_k)^2 \alpha_0^{H_k}(\vec{X},\vec{Q}), \tag{S6}$$

where $\alpha_0^{H_k} = Z_k(\vec{X},\vec{Q})^2/(m_{\mathrm{H}}\Omega_H^2)$. To derive Eq. (1) of the main text, we average over the direction of the external field. [It is enough to consider only three electric fields along the x,y, and z directions of the laboratory frame, and sum the three corresponding expressions from Eq. (S6).]

4. Spectroscopy

In the optical Kerr-effect setup, the probe pulses are near-infrared with wavelength λ = 1028nm and pulse duration τ = 270 fs; the pump pulses are variable-wavelength mid-IR, inducing birefringence at the probe sample after being delayed inside the test sample. The

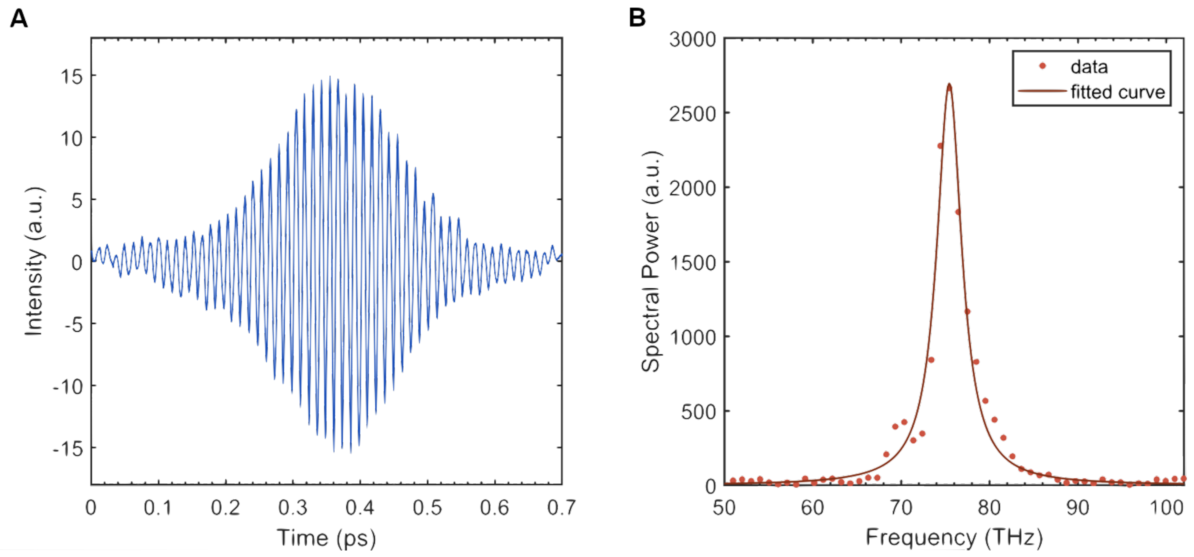

Figure S2: (A) Signal as collected from a Michelson interferometer, where the delay between the recombined mid-IR pump pulses is created via use of a translation stage. (B) Result of a fast Fourier transform of the signal in (A), showing clearly the frequency spectrum. The solid line is the Lorentizan fit of the data, from which we extract the central wavelength and full width half maximum (FWHM).

pump beam is modulated with an optical chopper at 750 Hz, and signal is detected via lock-in detection. The pump pulses are produced by an optical parametric amplifier (Light Conversion Orpheus) coupled to an amplified pulsed laser (Light Conversion Pharos). The wavelength for each measured datapoint is independently calibrated using Fourier-transform infrared spectroscopy (see the inset in Fig. 2B of the Main Text). For this step, the pump pulses and signal are locked-in at 131 Hz. We utilize a simple Michelson interferometer setup to determine the wavelength of the pump probe for each measured datapoint in the experiment. One example of such transform to obtain the wavelength is shown in Fig. S2.

The data in Fig. 2B of the main text can also be used to calculate the extinction coefficient, κ: neglecting the Fresnel losses at the perovskite surface in comparison to bulk absorption, κ can be found from the ratio of the intensity transmitted through $\mathbf{T}$ to the incident intensity. The ratio of transmission with (I_{with}) and without (I_{without}) the test sample (given that the attenuation of light with distance z is exponential, $I = \exp\left\{-\frac{4\pi\kappa z}{\lambda_0}\right\}$) is given

by

$$\frac{I_{\text{with}}}{I_{\text{without}}} = \frac{\exp\left(-4\pi\kappa_{\text{air}}{}^{(z-d)}/\lambda_0\right)\exp\left(-4\pi\kappa\,{}^{d}/\lambda_0\right)}{\exp\left(-4\pi\kappa_{\text{air}}\,{}^{z}/\lambda_0\right)} \quad\rightarrow\quad \kappa = -\ln\left[\frac{I_{\text{with}}}{I_{\text{without}}}\right]\times\frac{\lambda_0}{4\pi d}, \tag{S7}$$

where d is the sample thickness, λ_0 is the wavelength of the pump in vacuum, and we have assumed that the extinction coefficient of air, κ_{air} is 0.

5. Extinction coefficient

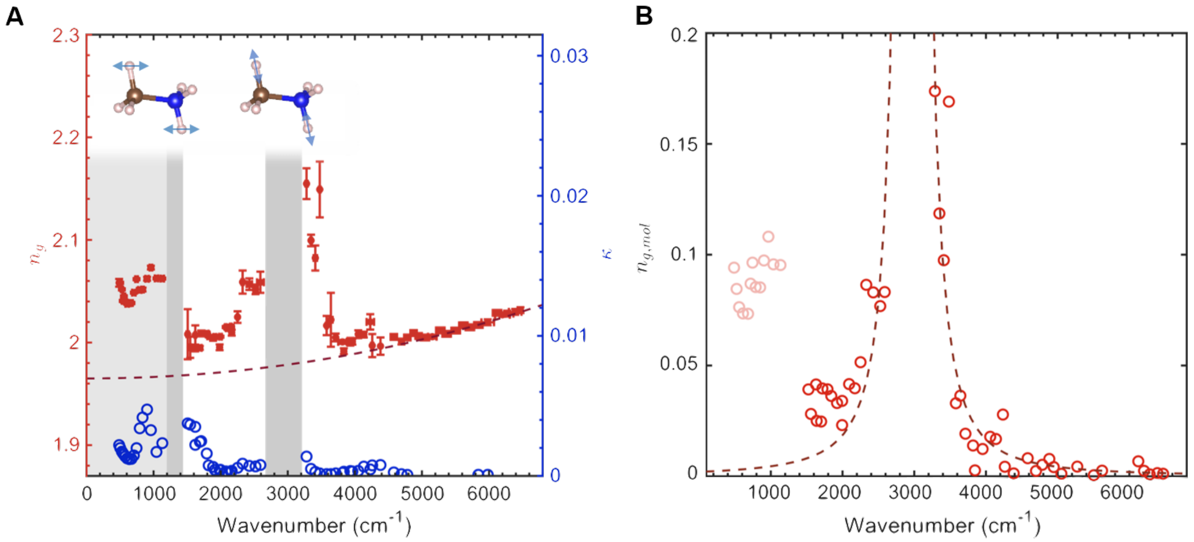

Figure S3: Extinction data appended on the data as presented in the main text, see Fig. 3 of the main text. The red dashed line is the electronic group index obtained from the Sellmeier fit to high energy data of Ref.[13]. The two prominent regions of vibrational resonance are indicated by the grey shading. Cartoon depictions of these families of vibrational modes are presented above the shaded regions, with the higher energy modes indicating C(N)–H stretching and the lower energy modes C(N)-H bending. Only one C(N)-H bond's direction of motion (toward against the bond) is shown with a blue arrow, for clarity, representing the entire cluster of modes in that energy region. (B) Molecular contribution to the group index, $n_{\text{g,mol}}$, around the region of stretching modes in (A) and Fig. 3 of the main text, obtained through subtracting the electronic component from the Sellmeier fit. The dashed line is the resultant fit using Eq. (2) of the main text.

Figure S3A shows the obtained extinction together with the data from Fig. 3 of the main text. The missing values in the regions of resonance are a result of strong absorption of the

sample. The low value of κ in the region outside the resonance suggests that these modes (clustered near a single resonance energy) are relatively strong and can be approximately considered in isolation from the other modes. Indeed, upon subtracting the electronic cage contribution to the refractive index from the high-energy Sellmeier fit to our previous data[13] as depicted in Fig. S3B, we achieve an excellent fit with Eq. (2) of the main text, which is derived for a single resonance energy $\hbar\Omega_H$.

6. Derivation of Eq. (2) of the main text

We use the Lorentz-Lorenz relation between the phase refractive index n and the polarizabilities of all degrees of freedom of the system $\{\alpha_i\}$[14]:

$$\frac{n_{\text{ph}}^2 - 1}{n_{\text{ph}}^2 + 2} = \sum_i \frac{4\pi N}{3} \alpha^i(\omega),$$

where N is the number density of atoms and the sum goes over all degrees of freedom including electrons. As can be seen in Fig. 3 of the main text, the molecular contribution to the total phase refractive index $n_{\text{ph}} = n_{\text{ph,el}} + n_{\text{ph,mol}}$ is small. Therefore, $n_{\text{ph,mol}}$ and the molecular polarizability α_{mol}^i are related as

$$\frac{6n_{\text{ph,el}}}{(n_{\text{ph,el}}^2 + 2)^2} n_{\text{ph,mol}} \approx \sum_{i\in\{\text{mol}\}}' \frac{4\pi N}{3} \alpha_{\text{mol}}^i(\omega), \tag{S8}$$

where the sum goes over molecular degrees of freedom. The polarizability of the C(N)–H stretching mode in the vicinity of its resonant frequency Ω_H diverges as $\alpha_{\text{mol}}^H(\omega) \approx \langle\alpha_0^H\rangle\Omega_H^2/(\Omega_H^2 - \omega^2)$ with $\langle\alpha_0^H\rangle$ being the average static (longitudinal) polarizability of the C(N)–H bond. Therefore, we can further simplify Eq. (S8) by keeping only the term corresponding to the mode in question.

We convert the phase index $n_{\text{ph,mol}} = n_{\text{ph}} - n_{\text{ph,el}}$ into the group index $n_{\text{g,mol}} \approx \partial(\omega n_{\text{ph,mol}})/\partial\omega$,

and re-write Eq. (S8)

$$n_{\mathrm{g,mol}}(\omega) \approx \langle \alpha_0^H \rangle \frac{4\pi N}{3} \frac{(\bar{n}_{\mathrm{el}}^2+2)^2}{12\bar{n}_{\mathrm{el}}} \cdot \frac{\Omega_H^2}{(\Omega_H-\omega)^2}, \tag{S9}$$

where $\bar{n}_{\mathrm{el}} \equiv n_{\mathrm{ph,el}}(\omega=\Omega_H)$. $N = \frac{1\,\mathrm{molecule}}{(5.92\mathrm{\AA})^3}$ in our calculations (the lattice constant $a = 5.92\mathrm{\AA}$ is taken from Ref.[15], see also[16,17]).

7. Uncorrelated dynamics

If the cage and the molecule are uncorrelated, we write

$$\langle \alpha_0^H \rangle_{\mathrm{uncorr}} = \frac{1}{3} \sum_{H_k=1}^{6} \int \mathrm{d}\vec{X}\, d\vec{Q} \rho_{\mathrm{mol}}(\vec{X}) \rho_{\mathrm{cage}}(\vec{Q})\, \alpha_0^{H_k}(\vec{X},\vec{Q}), \tag{S10}$$

where $\rho_{\mathrm{mol}}(\vec{X})$ $[\rho_{\mathrm{cage}}(\vec{Q})]$ is the density matrix that describes the molecule [the cage]. As there are (by assumption) no correlations between the lattice and MA, we place the molecule in the middle of the lattice unit, and assume that all allowed positions of the C–N and C(N)–H bonds are equally likely. Furthermore, we assume that $\alpha_0^{H_k}(\vec{X},\vec{Q}) \simeq \alpha_0^{H_k}(\vec{X},\vec{Q}_0) + \vec{c}(\vec{Q}-\vec{Q}_0)$, where $\vec{Q}_0$ are average positions of the atoms in the cage and $\vec{c}$ is the vector of expansion coefficients. [Note that this assumption is not necessary for our derivation as will become clear below.] This assumption is natural, as the atoms in the cage can move only around their equilibrium positions with a relatively small amplitude. Furthermore, it simplifies the derivation because (for harmonic dynamics of the cage) the linear term $(\vec{Q}-\vec{Q}_0)$ disappears.

With this, we write

$$\langle \alpha_0^H \rangle_{\mathrm{uncorr}} = \int \mathrm{d}\vec{X} \rho_{\mathrm{mol}}(\vec{X})\, \alpha_0^{H_{\mathrm{N}}}(\vec{X},\vec{Q}_0) + \int \mathrm{d}\vec{X} \rho_{\mathrm{mol}}(\vec{X})\, \alpha_0^{H_{\mathrm{C}}}(\vec{X},\vec{Q}_0), \tag{S11}$$

where H_{N} (H_{C}) refers to a hydrogen atom from the N–H (C–H) bond. We assume that the longitudinal polarizability depends only on $\vec{n}\vec{E}_{\vec{Q}_0}(\vec{X}_H)$, i.e., $\alpha_0^H(\vec{X},\vec{Q}_0) = \alpha_0^H(\vec{n}\vec{E}_{\vec{Q}_0}(\vec{X}_H))$ where $\vec{E}_{\vec{Q}_0}(\vec{X}_H)$ is the crystal field at the locus of the C(N)-H bond. This assumption is

based on (i) the observation that the fields in the center of the lattice unit of a cubic lattice are weak (they cancel each other due to the symmetry of the unit), and (ii) on the results of our DFT calculations. Further physical intuition can be gained by using a charged ellipsoid model for the C(N)–H bond.

Since the fields in the center of the lattice unit of a cubic lattice are weak, we write

$$\langle \alpha_0^H \rangle_{\text{uncorr}} = \int \mathrm{d}\vec{X} \rho_{\text{mol}}(\vec{X}) [\alpha_0^{H_\text{N}}(\vec{E}=0) + \alpha_0^{H_\text{C}}(\vec{E}=0) + C_{H_\text{C}} \vec{n} \vec{E}_{\vec{Q}_0}(\vec{X}_{H_\text{C}}) + C_{H_\text{N}} \vec{n} \vec{E}_{\vec{Q}_0}(\vec{X}_{H_\text{N}})], \tag{S12}$$

where the expansion coefficients C_{H_k} do not depend on the electric field. The integral $\int \mathrm{d}\vec{X} \rho_{\text{mol}}(\vec{X}) \vec{n} \vec{E}_{\vec{Q}_0}(\vec{X}_H)$ vanishes. To see this, we expand the vector $\vec{n}$ in components parallel and perpendicular to $\vec{R}$ (for a sketch of the geometry, see Fig. 1 of the main text). The two resulting integrals $\int \mathrm{d}\vec{X} \rho_{\text{mol}}(\vec{X}) \vec{n}_{\text{par}} \vec{E}_{\vec{Q}_0}(\vec{X}_H)$ and $\int \mathrm{d}\vec{X} \rho_{\text{mol}}(\vec{X}) \vec{n}_{\text{perp}} \vec{E}_{\vec{Q}_0}(\vec{X}_H)$ vanish due to Gauss's law and Kirchhoff's loop rule, respectively, which allows us to derive the expression presented in the main text:

$$\langle \alpha_0^H \rangle_{\text{uncorr}} = \alpha_0^{H_\text{N}}(\vec{E}=0) + \alpha_0^{H_\text{C}}(\vec{E}=0). \tag{S13}$$

8. Correlated dynamics

To estimate the value of the polarizability in case of correlated dynamics, we approximate the density matrix as

$$\rho(\vec{X}, \vec{Q}) = \rho_{\text{mol}}(\vec{X}; \vec{Q}_0) \rho_{\text{cage}}(\vec{Q}), \tag{S14}$$

where $\rho_{\text{mol}}(\vec{X}; \vec{Q}_0)$ describes the molecule assuming that the atoms in the cage are located at their average positions, $\vec{Q}_0$; $\rho_{\text{cage}}(\vec{Q})$ describes the atoms in the cage. Equation (S14) relies on the observation that the time scale for the cage dynamics is sub-ps whereas the dynamics of the C–N reorientation is 'much' longer, on the order of a few ps[18–20].

We assume that $\alpha_0^{H_k}(\vec{X}, \vec{Q}) \simeq \alpha_0^{H_k}(\vec{X}, \vec{Q}_0) + \vec{c}(\vec{Q} - \vec{Q}_0)$ and that the dynamics of the cage

is harmonic, which allows us to write

$$\langle \alpha_0^H \rangle \simeq \frac{1}{3} \int \mathrm{d}\vec{X} \rho_{\mathrm{mol}}(\vec{X}; \vec{Q}_0) \sum_k \alpha_0^{H_k}(\vec{X}, \vec{Q}_0). \tag{S15}$$

To establish the form of $\rho_{\mathrm{mol}}(\vec{X}; \vec{Q}_0)$, we note that there are six degenerate positions of the C–N bond in the three (parallel to the surfaces) planes of symmetry of a cube, see, e.g., [18]. The interaction between the N–H bonds and the halide atoms in the cage determines the positions of the hydrogen atoms[16,21,22]. For simplicity, we assume that the complex N–H–Br forms a straight line, so that we can directly use our DFT results presented in Fig. 1 of the main text. This assumption is a crude approximation of the molecular position as the C(N)–H bond features a rapid 'wobbling-within-a-cone' motion[18], for review see[23]. However, with this, we can provide an intuitive illustration of the approach (which is the main purpose of our work), and, furthermore, to put a lower bound on the electric field felt by a molecule. Finally, we derive

$$\langle \alpha_0^H \rangle = \frac{1}{3 m_{\mathrm{H}} \Omega_H^2} \sum_k Z_k^2 (\vec{E}_{\vec{Q}_0}(\vec{X}_{H_k}) \cdot \vec{n}_k), \tag{S16}$$

where $\vec{E}_{\vec{Q}_0}(\vec{X}_{H_i})$ is the crystal field at the locus of the C(N)–H bond. Using this expression and Fig. 1 of the main text, we estimate $\vec{E}_{\vec{Q}_0}(\vec{X}_{H_i})$ whose value is reported in the main text. [To include the uncertainty in the position of Ω_H one could use $e^2/(m_{\mathrm{H}} \Omega_H^2)$ in the range $0.41 - 0.44$ Å^3.]

9. Coulomb fields inside the lattice

In HOIPs, molecules are located close to the centers of lattice units, and experience weak electric fields. To estimate these fields, we calculate the electric potential close to the center of a lattice unit assuming that Br and Pb are point particles with charges -1|e| and +2|e|,

respectively. This potential can be calculated from the Taylor series whose first terms read

$$\phi(\vec{x}) = \phi(0) + e\vec{x}\sum_{\mathrm{Br}_k} \frac{\vec{Q}_{\mathrm{Br}_k}}{|\vec{Q}_{\mathrm{Br}_k}|^3} - 2e\vec{x}\sum_{\mathrm{Pb}_k} \frac{\vec{Q}_{\mathrm{Pb}_k}}{|\vec{Q}_{\mathrm{Pb}_k}|^3} - e\sum_{\mathrm{Br}_k} \frac{3(\vec{Q}_{\mathrm{Br}_k}\vec{x})^2 - x^2 Q^2_{\mathrm{Br}_k}}{|\vec{Q}_{\mathrm{Br}_k}|^5} + 2e\sum_{\mathrm{Pb}_k} \frac{3(\vec{Q}_{\mathrm{Pb}_k}\vec{x})^2 - x^2 Q^2_{\mathrm{Pb}_k}}{|\vec{Q}_{\mathrm{Pb}_k}|^5} + \ldots , \tag{S17}$$

where $\vec{Q}_{\mathrm{Br}_k}$ ($\vec{Q}_{\mathrm{Pb}_k}$) is the position of the kth Br (Pb) atom, and the sum goes over all atoms of a lattice unit. Due to the cubic symmetry of the lattice, the sums presented in Eq. (S17) vanish, and one needs to consider higher-order terms in the expansion, which lead to weak electric fields for small values of x.

From the symmetry argument, one can expect that the electric fields behave as $|\vec{E}| \sim ex^3/(a/2)^5$ close to the center of the unit cell, here $a = 5.92$Å is the lattice constant[15]. [It is worthwhile noting that a fast decay of the electric field, $\sim 1/|\vec{Q}|^5$ is the reason why it is enough to consider a single lattice unit for our estimation.] Assuming that $x \simeq 1$Å, we obtain $|\vec{E}| \sim 0.06$ V/Å. [Numerical calculations give even a smaller value, and confirm the order of magnitude of this estimate.] This value is too small to account for the measured value of the polarizability. Therefore, we conclude that one cannot consider ions in the cage as point-like objects. The simplest extension consistent with cubic symmetry of the lattice is a quadruple instead of Pb–Br–Pb complex. This leads to the potential $\phi \sim \frac{D}{2d^3}$ at the locus of the N–H bond, here D is the quadrupolar moment and $d = 2.5$ Å (see the main text). We put an estimate $D \in [0.5, 1.6]$ eÅ^2 – a natural atomic value – by comparing to the electric field deduced from renormalization of the polarizability. In the main text, we report only the central value.

10. Hydrogen Bond

We estimate H-bonding interactions from the energy required to move the molecule to the center of the cage. Using our estimate of D, we find that this energy per a hydrogen bond

is $ZD/(2d^3) - ZD/(2d_1^3) \sim 0.1$ eV, where $d_1 \simeq 3.2$ Å, and we use the Born charge $Z = 0.4$e to represent the charge of the N–H hydrogen, see Fig. 1 of the main text. Although, this estimate includes a number of approximations, it agrees well with the values reported in the literature, supporting our conclusion regarding usefulness of bond spectroscopy.

References

(1) Saidaminov, M. I.; Abdelhady, A. L.; Murali, B.; Alarousu, E.; Burlakov, V. M.; Peng, W.; Dursun, I.; Wang, L.; He, Y.; Maculan, G. et al. High-quality bulk hybrid perovskite single crystals within minutes by inverse temperature crystallization. *Nat. Commun.* **2015**, *6*, 8586.

(2) Saidaminov, M. I.; Abdelhady, A. L.; Maculan, G.; Bakr, O. M. Retrograde solubility of formamidinium and methylammonium lead halide perovskites enabling rapid single crystal growth. *Chem. Commun.* **2015**, *51*, 17658–17661.

(3) Neese, F. The ORCA program system. *Wiley Interdiscip. Rev.: Comput. Mol. Sci.* **2012**, *2*, 73–78.

(4) Becke, A. D. Density-functional thermochemistry. III. The role of exact exchange. *J. Chem. Phys.* **1993**, *98*, 5648–5652.

(5) Lee, C.; Yang, W.; Parr, R. G. Development of the Colle-Salvetti correlation-energy formula into a functional of the electron density. *Phys. Rev. B* **1988**, *37*, 785–789.

(6) Dunning, T. H. Gaussian basis sets for use in correlated molecular calculations. I. The atoms boron through neon and hydrogen. *J. Chem. Phys.* **1989**, *90*, 1007–1023.

(7) Kendall, R. A.; Dunning, T. H.; Harrison, R. J. Electron affinities of the first-row atoms revisited. Systematic basis sets and wave functions. *J. Chem. Phys.* **1992**, *96*, 6796–6806.

(8) Leguy, A. M. A.; Goñi, A. R.; Frost, J. M.; Skelton, J.; Brivio, F.; Rodríguez-Martínez, X.; Weber, O. J.; Pallipurath, A.; Alonso, M. I.; Campoy-Quiles, M. et al. Dynamic disorder, phonon lifetimes, and the assignment of modes to the vibrational spectra of methylammonium lead halide perovskites. *Phys. Chem. Chem. Phys.* **2016**, *18*, 27051–27066.

(9) Pérez-Osorio, M. A.; Milot, R. L.; Filip, M. R.; Patel, J. B.; Herz, L. M.; Johnston, M. B.; Giustino, F. Vibrational Properties of the Organic–Inorganic Halide Perovskite $CH_3NH_3PbI_3$ from Theory and Experiment: Factor Group Analysis, First-Principles Calculations, and Low-Temperature Infrared Spectra. *J. Phys. Chem. C* **2015**, *119*, 25703–25718.

(10) Pérez-Osorio, M. A.; Lin, Q.; Phillips, R. T.; Milot, R. L.; Herz, L. M.; Johnston, M. B.; Giustino, F. Raman Spectrum of the Organic–Inorganic Halide Perovskite $CH_3NH_3PbI_3$ from First Principles and High-Resolution Low-Temperature Raman Measurements. *J. Phys. Chem. C* **2018**, *122*, 21703–21717.

(11) Gough, K. M. Theoretical analysis of molecular polarizabilities and polarizability derivatives in hydrocarbons. *J. Chem. Phys.* **1989**, *91*, 2424–2432.

(12) Le Févre, R. In *Molecular Refractivity and Polarizability*; Gold, V., Ed.; Advances in Physical Organic Chemistry; Academic Press, 1965; Vol. 3; pp 1–90.

(13) Volosniev, A. G.; Shiva Kumar, A.; Lorenc, D.; Ashourishokri, Y.; Zhumekenov, A. A.; Bakr, O. M.; Lemeshko, M.; Alpichshev, Z. Spin-Electric Coupling in Lead Halide Perovskites. *Phys. Rev. Lett.* **2023**, *130*, 106901.

(14) Born, M.; Wolf, E. *Principles of Optics*; Cambridge University Press, 2019.

(15) Weber, D. $CH_3NH_3PbX_3$, ein Pb(II)-System mit kubischer Perowskitstruktur / $CH_3NH_3PbX_3$, a Pb(II)-System with Cubic Perovskite Structure. *Z. für Naturforsch. B* **1978**, *33*, 1443–1445.

(16) Yin, T.; Fang, Y.; Fan, X.; Zhang, B.; Kuo, J.-L.; White, T. J.; Chow, G. M.; Yan, J.; Shen, Z. X. Hydrogen-Bonding Evolution during the Polymorphic Transformations in $CH_3NH_3PbBr_3$: Experiment and Theory. *Chem. Mater.* **2017**, *29*, 5974–5981.

(17) Abia, C.; López, C. A.; Cañadillas-Delgado, L.; Fernández-Diaz, M. T.; Alonso, J. A. Crystal structure thermal evolution and novel orthorhombic phase of methylammonium lead bromide, $CH_3NH_3PbBr_3$. *Sci. Rep.* **2022**, *12*, 18647.

(18) Leguy, A.; Frost, J.; McMahon, A.; et al. The dynamics of methylammonium ions in hybrid organic–inorganic perovskite solar cells. *Nat. Commun.* **2015**, *6*, 7124.

(19) Quarti, C.; Mosconi, E.; Ball, J. M.; D'Innocenzo, V.; Tao, C.; Pathak, S.; Snaith, H. J.; Petrozza, A.; De Angelis, F. Structural and optical properties of methylammonium lead iodide across the tetragonal to cubic phase transition: implications for perovskite solar cells. *Energy Environ. Sci.* **2016**, *9*, 155–163.

(20) Mattoni, A.; Filippetti, A.; Caddeo, C. Modeling hybrid perovskites by molecular dynamics. *J. Phys.: Condens. Matter* **2016**, *29*, 043001.

(21) Lee, J. H.; Lee, J.-H.; Kong, E.-H.; M., J. H. The nature of hydrogen-bonding interaction in the prototypic hybrid halide perovskite, tetragonal $CH_3NH_3PbI_3$. *Sci. Rep.* **2016**, *6*, 21687.

(22) Motta, C.; El-Mellouhi, F.; Sanvito, S. Exploring the cation dynamics in lead-bromide hybrid perovskites. *Phys. Rev. B* **2016**, *93*, 235412.

(23) Gallop, N. P.; Selig, O.; Giubertoni, G.; Bakker, H. J.; Rezus, Y. L. A.; Frost, J. M.; Jansen, T. L. C.; Lovrincic, R.; Bakulin, A. A. Rotational Cation Dynamics in Metal Halide Perovskites: Effect on Phonons and Material Properties. *J. Phys. Chem. Lett.* **2018**, *9*, 5987–5997.
